# Roles and mechanisms of leptin in osteogenic stimulation in cervical ossification of the posterior longitudinal ligament

**DOI:** 10.1186/s13018-018-0864-4

**Published:** 2018-07-03

**Authors:** Bin Feng, Shiliang Cao, Jiliang Zhai, Yi Ren, Jianhua Hu, Ye Tian, Xisheng Weng

**Affiliations:** Department of Orthopedics Surgery, Peking Union Medical College Hospital, Chinese Academy of Medical Sciences & Peking Union Medical College, Beijing, 100730 China

**Keywords:** Leptin, Ossification of the posterior longitudinal ligament (OPLL), Gender, Bone metabolic markers, Signaling pathway

## Abstract

**Background:**

Hyperleptinemia is a common feature of obese people, and leptin, an adipocyte-derived cytokine, is believed to be an important factor in the pathogenesis of cervical ossification of the posterior longitudinal ligament(C-OPLL). So this research was to identify the relation between the serum leptin and bone metabolic markers and how the leptin induced osteogenic effect in C-OPLL.

**Methods:**

Sixty-four samples were selected to determine the concentration of leptin, insulin, and alkaline phosphatase. And the association of leptin with these factors was also examined. We also evaluate the effect of leptin on the development of C-OPLL and further explored the possible underlying mechanism in vitro.

**Results:**

We found that serum leptin concentrations were higher in females than in males. Serum leptin and ALP concentrations were increased significantly in C-OPLL females compared to non-OPLL females. In OPLL subjects, the serum leptin concentration corrected for body mass index correlated negatively with the ALP concentrations. In C-OPLL cells, leptin treatment led to a significant increase in mRNA expressions of ALP and OCN and formation of mineralized nodule. Our experiments reported here that osteogenic effect of leptin in C-OPLL cells could be mediated via ERK1/2, p38 MAPK, and/or JNK signaling pathways.

**Conclusions:**

From this research, we got that leptin treatment led to a significant increase in mRNA expressions of ALP and OCN and formation of mineralized nodule. And the osteogenic effect of leptin in C-OPLL cells could be mediated via ERK1/2, p38 MAPK, and/or JNK signaling pathways.

## Background

Ossification of the posterior longitudinal ligament (OPLL) is a common musculoskeletal disease, characterized by ectopic bone formation of the spinal ligament preferentially at the cervical spine [1, 2]. As OPLL commonly involved in cervical spine resulting in myelopathy or radiculopathy, in this condition, we said it cervical OPLL(C-OPLL). Enlarged ossified ligament compresses the spinal cord and nerve roots, eventually leads to the neurological deficit [3, 4]. Its prevalence is higher among Asian populations, and in Chinese populations, the average prevalence is reported to be 3.08% [5–8].

Although the exact pathogenesis of C-OPLL remains unclear, leptin is supposed to be an important factor in the pathogenesis of C-OPLL. Leptin, a product of the ob gene which is expressed by adipocyte tissue and released into circulation, is a prominent regulator of body weight and fat [9, 10]. Investigations have indicated that leptin can stimulate the proliferation and osteogenic differentiation of embryonic cells, bone marrow stromal cells (BMSCs), and osteoblastic cells [11–13]. Leptin has been found to have both positive and negative effects on bone mass [14]. Leptin positively regulates bone formation through direct actions on bone, when administrated peripherally [10, 15], and suppresses bone formation and increases resorption through a hypothalamic relay, when infused centrally [16]. Previous study indicates that serum leptin may be associated with the development of heterotopic ossification of the spinal ligament [17]. However, the association between leptin and OPLL is still controversial and needs to be clarified further. And the mechanism of leptin in the development of OPLL has seldom been studied.

In this study, to clarify the association between leptin and C-OPLL, we measured serum leptin concentrations in C-OPLL patients and non-OPLL controls and corrected these levels using individual body mass index (BMI). Then, we analyzed the possible mechanism of leptin in the development of OPLL in vitro.

## Methods

### Patients

Totally, 64 samples were used for the research, including 35 cervical OPLL (24 males and 11 females) patients who underwent anterior cervical decompression surgery and 29 non-OPLL (15 males and 14 females) controls; the majority of who had cervical degenerative disorders were enrolled into this study. The diagnosis of cervical OPLL was confirmed by computer tomography (CT), X-ray photographs, and magnetic resonance imaging (MRI) of the cervical spine. This study was approved by the Ethics Committee of Peking Union Medical College Hospital, and informed consents were obtained from all patients.

Previous data indicated that there are important gender-based differences in the regulation and action of leptin in humans, and leptin levels are higher in women than in men. We subdivided the OPLL and non-OPLL groups according to gender. The mean age of OPLL males, non-OPLL males, OPLL females, and non-OPLL females was 59.04 ± 9.32, 57.40 ± 11.04, 57.25 ± 8.68, and 61.95 ± 10.93 years, respectively. The mean BMI (weight in kilograms divided by the square of height in meters) of the four groups was 26.47 ± 3.46, 25.26 ± 4.46, 25.49 ± 3.05, and 24.1 ± 3.37 kg/m^2^, respectively.

### Blood samples

Blood samples were collected from all patients between 8:00 and 10:00 after overnight fasting and the serum immediately frozen at − 80 °C until analysis. Serum leptin and insulin concentrations were measured using a commercially available enzyme-linked immunosorbent assay (ELISA) kit (Elabscience). The serum concentrations of bone formation markers, alkaline phosphatase (ALP), were measured using BCIP/NBT ALP Color Development Kit (Beyotime Biotechnology). The concentration of glycosylated serum protein (GSP), which can effectively reflect the average glucose concentration of the patients in the past 2~3 weeks, was also detected using GSP test kit (Solarbio life sciences).

### Spinal ligament samples

During the anterior cervical decompression surgery, posterior longitudinal ligament specimens were aseptically harvested from OPLL patients and rinsed with phosphate-buffered saline. Surrounding tissue was carefully removed under a dissecting microscope.

### Cell cultures

The collected ligaments were mincing into approximately 0.5 mm^3^ pieces and washed twice with phosphate-buffered saline (PBS). Then, the ligament fragments were plated into 6 cm culture dishes and maintained in low-glucose Dulbecco’s modified Eagle’s media (DMEM) (supplemented with 10% FBS, 1% L-glutamine, 100 units/ml of penicillin G sodium, 10 mM-glycerophosphate, and 100 μg/ml of streptomycin sulfate) in a humidified atmosphere of 95% air and 5% CO_2_ at 37 °C. The cells derived from the explants were removed from the dishes with 0.02% EDTA, 0.05% trypsin for further passage. The first to third passage cells were used in the following study.

### CCK8 assay

Cell proliferation was measured by CCK8 dye reduction assay. Briefly, 5 × 10^3^ cells were seeded into 96-well plates overnight and exposed to the leptin at distinct concentrations (0, 50, 100, 200, 400 ng/ml) for different times (4 and 7 days). Then, the cells were incubated with 10 μl of CCK8 for 1 h at 37 °C. The absorbance was measured at 450 nm using a microplate reader.

### Real-time PCR

3 × 10^6^ cells in logarithmic phase were plated into 6-well dishes for 24 h, then the cells were exposed to the leptin at different concentrations (0, 50, 100, 200 ng/ml). After 96 h, total RNA was extracted from the cells using Trizol reagents (Invitrogen), and the RNA concentration was detected. Two micrograms of total RNA were reverse-transcribed using Reverse Transcription Kit (Invitrogen) for real time-PCR (RT-PCR). Primers used for amplification were as follows: ALP, 5′-TCCCAGTTGAGGAGGAGAA-3′ (forward), 5′-CCAGGAAGATGATGAGGTTC-3′ (reverse); osteocalcin (OCN), 5′-AGCGAGGTAGTGAAGAGAC-3′ (forward), 5′-CCTGAAAGCCGATGTGGT-3′ (reverse); β-actin, 5′-ATCATGTTTGAGACCTTCAACA-3′(forward), 5′-CATCTCTTGCTCGAAGTCCA3′ (reverse). Polymerase chain reaction amplification was carried out in a volume of 25 μl containing 12.5 μl 2× PCR mix, 10.5 μl nuclease-free water, 1 μl cDNA, and 1 μl primer. The melting curves were also prepared during the amplifications. All products were normalized to β-actin mRNA levels. Each specimen was repeated three times.

### Mineralization assay

1 × 10^4^ cells were plated into 12-well dishes and maintained in DMEM with 10% FBS. On confluence, designated day 0, cells were exposed to leptin medium containing DMEM supplemented with 10% FBS, 1% L-glutamine, 100 units/ml of penicillin G sodium and 100 μg/ml of streptomycin sulfate, 10 mM-glycerophosphate, and 100 ng/ml leptin. Alizarin red assay (Sigma) was performed at 72 and 96 h to determine the mineralization. Briefly, cells were washed with D-hank’s and fixed with 4% paraformaldehyde for 20–30 min at room temperature. Fixed cultures were incubated with 1% alizarin red for 20–30 min at 37 °C and washed with distilled water for three times to remove the excessive dye. Extracellular matrix mineral-bound stains were visualized and photographed under a microscope.

### ALP activity assay

ALP activity was evaluated using commercially available kits. Cells were cultured in 6-well plates for 3–5 h and exposed to the leptin at different concentrations (0, 50, 100, 200 ng/ml). After 96 h, cells were washed with PBS and fixed in 4% polyoxymethylene for 10 min, and stained with BCIP/NBT ALP Color Development Kit (Beyotime Biotechnology) according to the manufacturer’s instructions.

### Western blotting

Cells were harvested, and equal amounts of protein were loaded onto 10% sodium dodecyl sulfate polyacrylamide gel electrophoresis (SDS–PAGE) gels for 2 h at 100 V and subsequently transferred onto polyvinylidene difluoride (PVDF) membranes (Millipore). The membrane was blocked with 5% skim milk for 1 h at room temperature. After washing three times with Tris-buffered saline (TBS) containing 0.1% Tween 20 (TBST), the membranes were incubated with appropriately diluted Phospho-p44/42 MAPK, p44/42 MAPK, Phospho-JNK/SAPK, JNK/SAPK, Phospho-p38 MAPK, and p38 MAPK (Beyotime, Shanghai, China) antibodies at 4 °C overnight. Then, the membranes were washed as before and incubated with horseradish peroxidase-conjugated secondary antibodies (anti-mouse or anti-rabbit) for 1 h at room temperature. After that, these membranes were washed thoroughly, to eliminate the unspecific antibody. At last, proteins were detected using enhanced chemiluminescence (ECL) blotting reagents according to the manufacturer’s instruction.

### Statistical analysis

Data was analyzed using mean ± SD. Student’s *t* test was used to compare the data between OPLL and non-OPLL groups. Tukey’s multiple comparison test was used to analyze the variance of the data and to estimate the level of significance. *p* < 0.05 was considered significant.

## Results

### Serum leptin concentrations and leptin/BMI ratios in OPLL and non-OPLL patients

As shown in Table 1, compared with the male subjects, both non-OPLL and OPLL groups had significantly higher serum leptin concentration in females. In females, serum leptin/BMI ratio in the OPLL group was 1.6-fold higher than that in the non-OPLL group (*p* < 0.05); meanwhile, serum concentrations of bone metabolism biomarkers ALP also increased significantly (*p* < 0.05). However, in male subjects, there was no obvious difference in serum leptin concentration between the OPLL and non-OPLL groups, and ALP concentrations showed no difference either. The serum insulin concentrations were higher in OPLL groups than that in non-OPLL groups in both female and male subjects. However, the difference was not statistically significant.

**Table 1:** Clinical characteristics of female and male OPLL and serum concentration of leptin, insulin, and ALP

Female OPLL versus non-OPLL			
	Non-OPLL (*n* = 14)	OPLL (*n* = 11)	*p* (Student’s *t*)
Age (year)	61.95 ± 10.93	57.25 ± 8.68	N.S.
Height (cm)	1.61 ± 0.05	1.62 ± 0.05	N.S.
Weight (kg)	62.79 ± 9.86	66.83 ± 10.55	N.S.
BMI (kg/m)	24.10 ± 3.37	25.49 ± 3.05	N.S.
Serum leptin (ng/ml)	22.30 ± 9.21	37.68 ± 25.22	< 0.05
Leptin/BMI	0.93 ± 0.33	1.50 ± 0.97	< 0.05
Serum insulin **(**μU/ml**)**	19.00 ± 8.60	22.90 ± 13.20	N.S.
Serum ALP	7.17 ± 1.11	8.05 ± 0.73	< 0.05
Male OPLL versus non-OPLL			
	Non-OPLL (*n* = 15)	OPLL (*n* = 24)	*p* (Student’s *t*)
Age (year)	57.4 ± 11.04	59.04 ± 9.32	N.S.
Height (cm)	170 ± 4.20	170 ± 7.07	N.S.
Weight (kg)	73.53 ± 13.68	76.61 ± 12.99	N.S.
BMI (kg/m)	25.26 ± 4.46	26.47 ± 3.46	N.S.
Serum leptin (ng/ml)	13.45 ± 6.32	16.69 ± 6.90	N.S.
leptin/BMI	0.52 ± 0.19	0.65 ± 0.25	N.S.
Serum insulin (μU/ml)	22.10 ± 11.00	29.20 ± 30.00	N.S.
Serum ALP	7.25 ± 1.57	7.97 ± 1.63	N.S.

### Correlation of leptin/BMI with serum insulin and biochemical maker, ALP

**Fig. 1:**
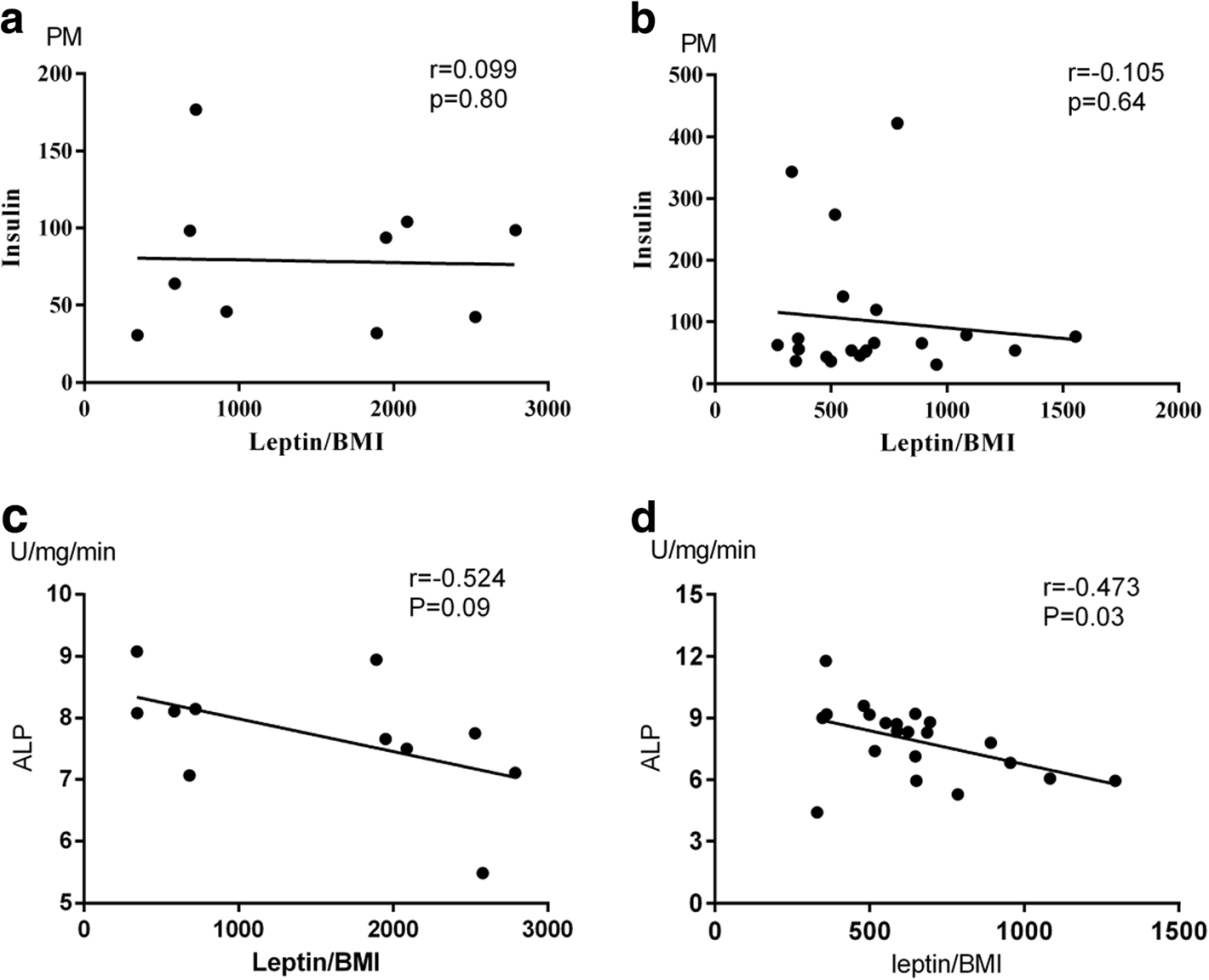
Relationship between the leptin/BMI (body mass index) ratio and serum insulin and biochemical maker, ALP concentrations with female and male OPLL patients. There was a negative, significant correlation between the leptin/BMI ratio and ALP (*p* < 0.05). **a**, **c** Females OPLL. **b**, **d** Males OPLL

To determine the factors associated with the leptin/BMI ratio in OPLL subjects, we examined the correlation between leptin/BMI ratios and serum insulin, ALP level (Fig. 1). There was only a relatively weak, non-significant positive correlation between the leptin/BMI ratio and serum insulin in females. In contrast, in male groups, the serum insulin concentration showed a weak, non-significant negative correlation with the leptin/BMI ratio. ALP concentrations were correlated negatively with the leptin/BMI ratio both in OPLL male groups and female groups, whereas male OPLL groups showed a significant correlation (*r* = − 0.473, *p* < 0.05). There was no significant correlation between the leptin/BMI ratios and serum insulin, ALP level, in non-OPLL subjects (data not shown).

### Effect of leptin on the proliferation of OPLL cells

**Fig. 2:**
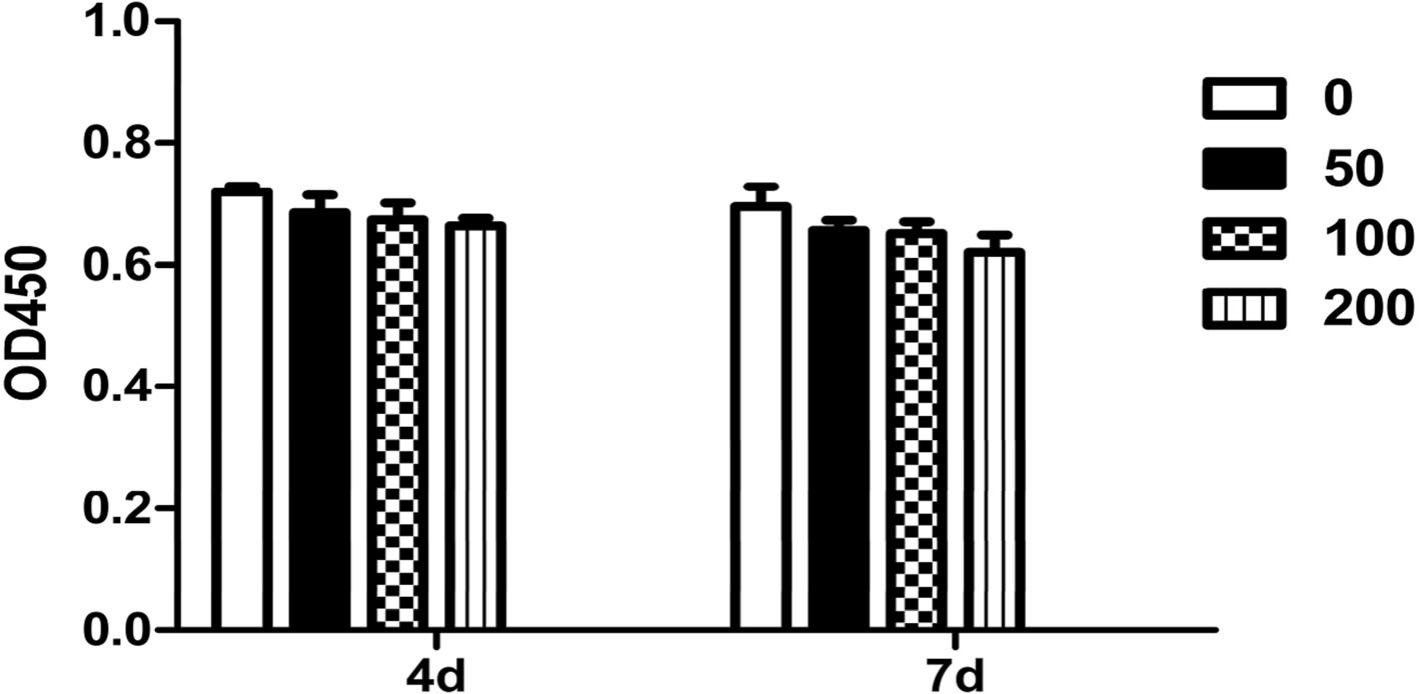
Effect of leptin on the proliferation of OPLL cells

To investigate the molecular mechanism underlying leptin-stimulated OPLL, the effect of leptin on cell proliferation of OPLL cells was evaluated by CCK8 method. As shown in Fig. 2, the leptin had no significant effect on the proliferation of OPLL cells with various leptin concentration (0, 50, 100, 200 ng/ml) at different periods of time (4 and 9 days).

### Effect of leptin on the osteogenic differentiation of OPLL cells

**Fig. 3:**
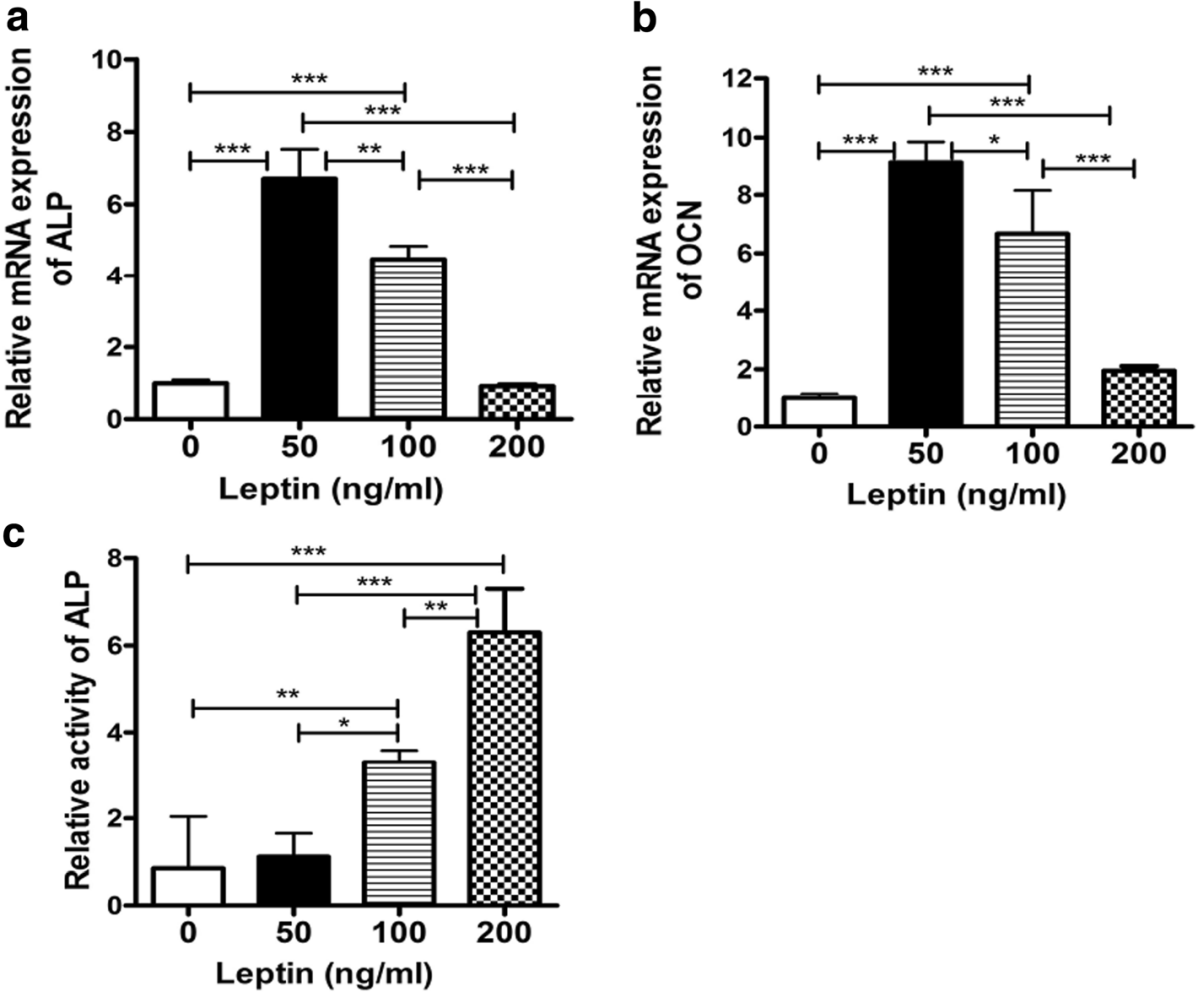
Effects of leptin on osteocalcin and ALP mRNA expressions and activity in OPLL cells. **a** ALP mRNA expression. **b** OCN mRNA expression. **c** ALP activity measurement. *p < 0.05; **p < 0.001; ***p < 0.0001

We then examined the osteogenic differentiation of OPLL cells by RT-PCR and ALP activity assay. RT-PCR analysis showed that leptin treatment resulted in a significant increase in mRNA expression of ALP and osteocalcin (OCN) in OPLL cells, and the effect was most obvious at 50 ng/ml leptin concentration (Fig. 3a, b). ALP activity assay demonstrated that the activity of ALP was significantly elevated in response to leptin stimulation in OPLL cells, and the effect was dose-dependent (Fig. 3c).

**Fig. 4:**
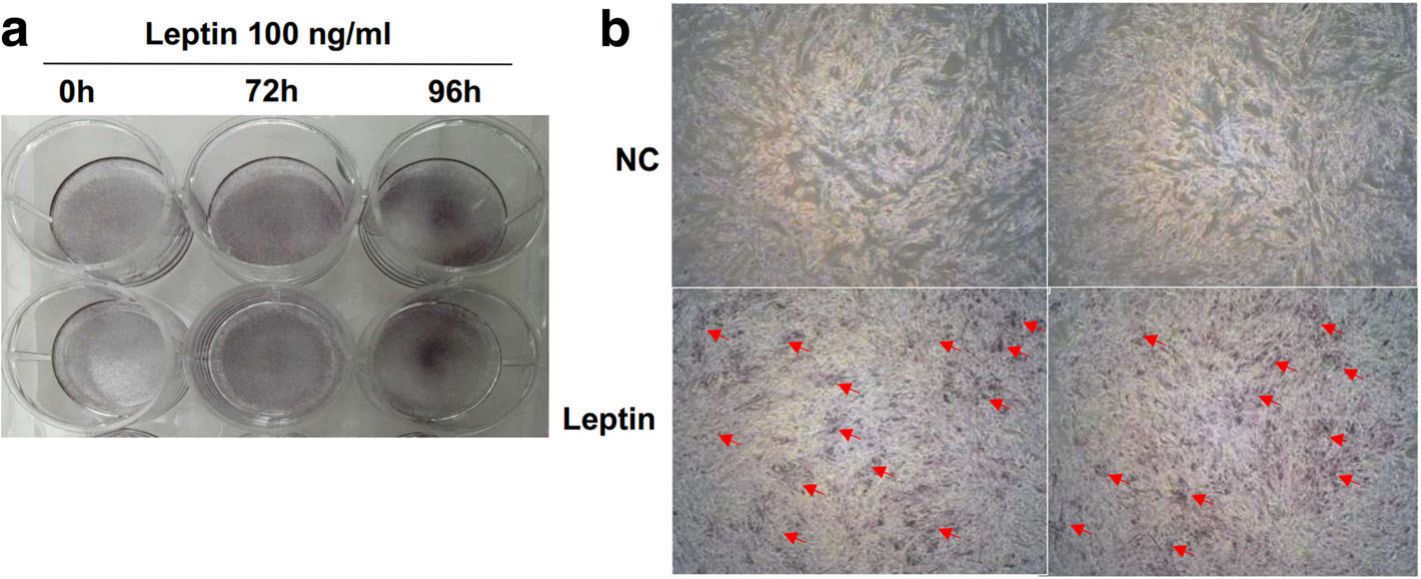
Effects of leptin on the formation of mineralized nodules in OPLL cells. **a** Different time points of leptin stimulation. **b** Leptin (100 ng/ml) stimulated for 96 h. Mineralized nodules are indicated by red arrows in the figure. NC, negative control

Apart from makers of osteogenic differentiation, we further examined the formation of mineralized nodules by alizarin red staining. Mineralization assays showed that under 100 ng/ml leptin stimulation, the cell matrix began to mineralize, and crystals appeared at 72 h. The mineralized nodules increased significantly at 96 h (Fig. 4a, b indicated by red arrows), compared with the absence of leptin treatment OPLL cells. There was no mineralization observed in the negative control (NC) group.

### Pathways involved in the leptin-stimulated osteogenic differentiation of OPLL cells

**Fig. 5:**
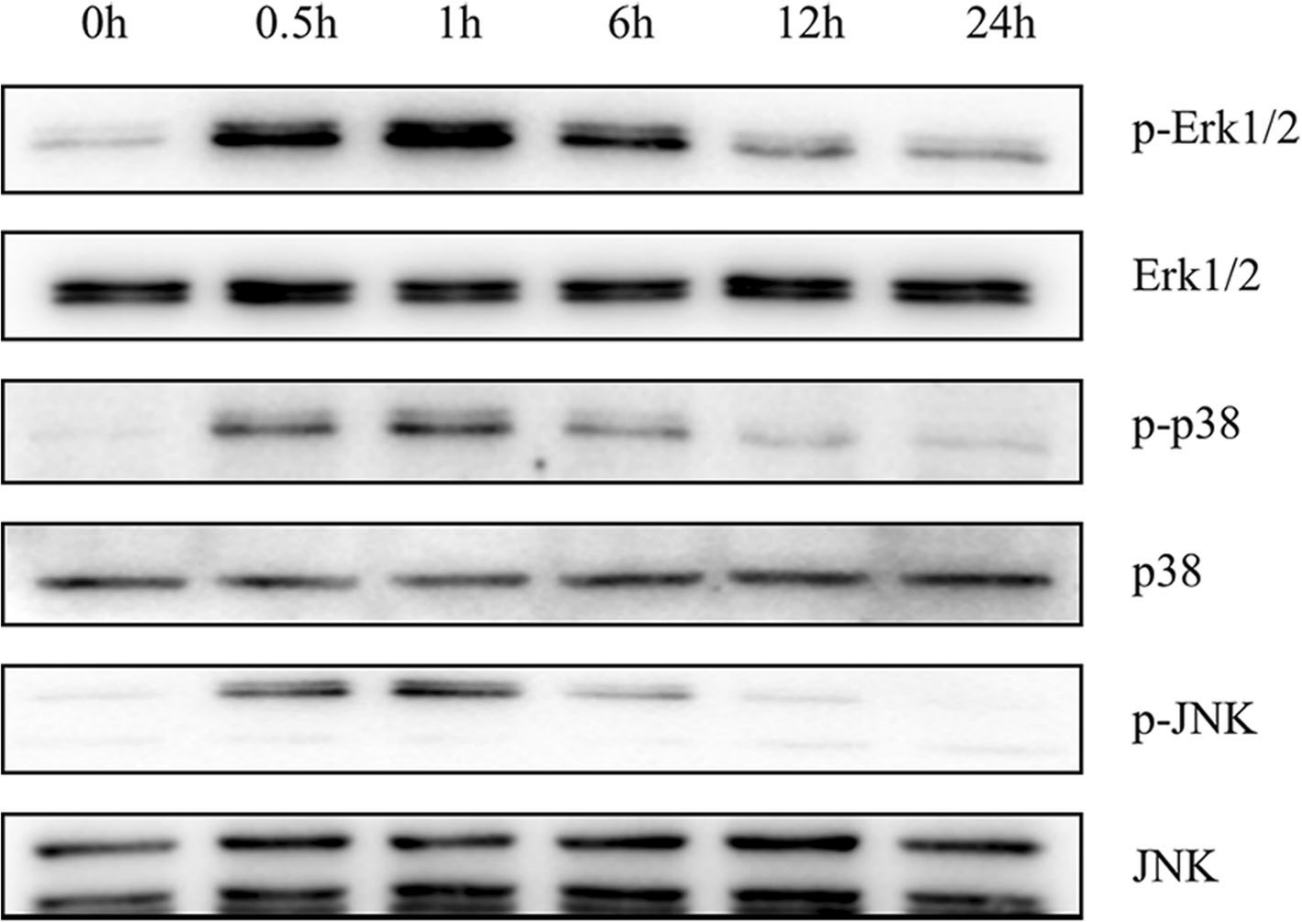
Activation of ERK1/2, p38MAPK, and JNK signaling pathway in OPLL cells after 100 ng/ml leptin treatment

To learn more about the mechanism underlying the osteogenic effect of leptin, we further examined the status of ERK, JNK, and p38 phosphorylation in response to leptin treatment in OPLL cells. Proteins were extracted from OPLL cells at different time after 100 ng/ml leptin treatment. The immunoblot results showed that leptin stimulated the phosphorylation of ERK1/2, p38 MAPK, and JNK in a time-dependent manner and the effect was most obvious at 1 h, while the total expression levels of ERK1/2, p38 MAPK, and JNK were unchanged over the time of leptin treatment (Fig. 5).

## Discussion

Many investigations have indicated that leptin is involved in many bone diseases [18–21]. OPLL is a common bone disease caused by heterotopic bone formation of the posterior longitudinal ligament. Obesity is considered to be the major risk factor of OPLL [5, 22]. Recent studies showed that hyperleptinemia, a common feature of obese people, was closely correlated with OPLL [18]. These studies suggest that leptin, an adipocyte-derived cytokine, plays a critical role in connecting at molecular levels the phenotypical manifestation of obesity and the pathological development of OPLL.

In this study, we firstly determined the association between serum leptin concentration and bone metabolic markers in patients with OPLL. Our results demonstrated that female subjects had significantly higher serum leptin concentration compared with male subjects, in both OPLL and non-OPLL groups. Serum leptin/BMI ratio in the OPLL groups was higher than that in the non-OPLL groups (*p* < 0.05) in female subjects. However, there was no obvious difference in the male subjects. Further analysis indicated that the leptin/BMI ratio correlated negatively with the ALP concentrations in both OPLL male and female groups, whereas male groups showed a significant correlation (*r* = − 0.473, *p* < 0.05). Nevertheless, there were only 11 subjects, and the individual difference was great, which resulted in no statistically significant difference. This result suggests that leptin can inhibit bone formation in vivo, which was in accordance with the report of Elefteriou et al. [23], which demonstrated in animal experiments that leptin was a determinant of bone formation and leptin anti-osteogenic function was conserved in vertebrates. We also found that in the female group, the leptin/BMI ratio was weakly correlated positively with serum insulin levels in female OPLL, whereas the negative correlation was observed in male OPLL group, which was consistent with the previous studies [17, 24, 25].

To further validate the role of leptin in OPLL, we performed OPLL cell differentiation experiments in vitro. And the cell experiments were derived from multiple individuals. We found that leptin treatment can lead to a significant increase in mRNA expressions of ALP and OCN in OPLL cells; the effect was most significant at 50 ng/ml leptin concentration. ALP activity assay demonstrated that the activity of ALP was significantly elevated in response to leptin stimulation in a dose-dependent manner in OPLL cells. Furthermore, leptin can induce the cell matrix mineralization, nodules, and crystals in OPLL cells, whereas it had no appreciable effect on the proliferation of OPLL cells. Our findings prove that leptin plays an important role in osteogenic differentiation and mineralization of OPLL cells, which is consistent with the previous reports [26–28] that peripheral leptin was essential for normal bone resorption and enhancement of bone formation.

At the same time, in this study, we try to confirm the molecular mechanism involved in leptin-stimulated osteogenesis in OPLL cells. It is believed that leptin exerted its biological function through binding to its receptors, which in turn transduced the signal through the activation of specific pathways. Previous studies indicated that leptin can activate many signaling pathways involving the JK/signal transducer and activator of transcription (JAK/STAT), as well as PI3-K and MAPK, to regulate chondrocyte differentiation [29–33]. However, the involvement in leptin induction of osteogenic differentiation has not been studied. In our study, we found that leptin increased the expressions of ALP and OCN in a dose-dependent manner and the formation of mineralized nodule. At the same time, the phosphorylation of ERK1/2, JNK, and p38MAPK was activated by leptin. In summary, these results indicated that ERK1/2, JNK, and p38MAPK might be the signaling pathways mediating leptin-stimulated osteogenic differentiation in OPLL cells. This was helpful for the further studies on investigating the molecular mechanism underlying osteogenic commitment of OPLL cells.

The main limitation in this study was that the sample size was small, especially in the OPLL female group, which resulted in no statistically significant difference of the serum insulin concentrations between OPLL and non-OPLL females, as well as no significant correlations between leptin and both insulin and ALP. Nevertheless, even with the limitation of the sample size, in this study, our results were consistent with the results of preceding studies.

In conclusion, we found that leptin may negatively regulate bone formation in vivo, through a central hypothalamic relay, whereas positively promoted the osteogenic differentiation in vitro through the peripheral pathway as previous studies report [23, 28, 34]. Furthermore, activated ERK1/2, JNK, and p38MAPK signaling pathways might mediate leptin-stimulated osteogenic differentiation in OPLL cells. The results of our research may have significant enlightenment in understanding the mechanisms of spinal ligament growth. And further studies are needed to confirm our findings and to evaluate other possible mechanisms involved.

## Conclusions

From this research, we found that serum leptin concentration was higher in female subjects compared with male subjects in both C-OPLL and non-OPLL groups. Serum leptin and ALP concentrations increased significantly in C-OPLL female compared to non-OPLL female. In both male and female with OPLL, the serum leptin concentration corrected for BMI correlated negatively with the ALP concentrations. In C-OPLL cells, leptin treatment led to a significant increase in mRNA expressions of ALP and OCN and the formation of mineralized nodule. The osteogenic effect of leptin in C-OPLL cells might be mediated via ERK1/2, p38 MAPK, and/or JNK signaling pathways.

## Abbreviation

ALP: Alkaline phosphatase activity
CCK-8: Cell Counting Kit-8
C-OPLL: Cervical OPLL
DMEM: Dulbecco’s modified Eagle’s medium
ERK: Extracellular signal-regulated kinase
FBS: Fetal bovine serum
GSP: Glycosylated serum protein
JNK: c-Jun NH2-terminal kinase
MAPK: Mitogen-activated protein kinase
OCN: Osteocalcin
OPLL: Ossification of the posterior longitudinal ligament
